# Influenza hospitalization epidemiology from a severe acute respiratory infection surveillance system in Jordan, January 2008–February 2014

**DOI:** 10.1111/irv.12354

**Published:** 2016-01-29

**Authors:** Mohammad Al‐Abdallat, Patrick Dawson, Aktham Jeries Haddadin, Waleed El‐Shoubary, Erica Dueger, Tarek Al‐Sanouri, Mayar M. Said, Maha Talaat

**Affiliations:** ^1^ Ministry of HealthAmmanJordan; ^2^ Global Disease Detection Center, U.S. Centers for Disease Control and PreventionCairoEgypt; ^3^ U.S. Naval Medical Research Unit No. 3CairoEgypt

**Keywords:** Acute respiratory infections, influenza, influenza seasonality, Jordan, severe acute respiratory infections

## Abstract

**Background:**

Acute respiratory infections (ARIs) are a major cause of morbidity and mortality worldwide. Influenza typically contributes substantially to the burden of ARI, but only limited data are available on influenza activity and seasonality in Jordan.

**Methods:**

Syndromic case definitions were used to identify individuals with severe acute respiratory infections (SARI) admitted to four sentinel hospitals in Jordan. Demographic and clinical data were collected. Nasopharyngeal and oropharyngeal swabs were tested for influenza using real‐time reverse transcription polymerase chain reaction and typed as influenza A or B, with influenza A further subtyped.

**Results:**

From January 2008–February 2014, 2891 SARI cases were tested for influenza, and 257 (9%) were positive. While 73% of all SARI cases were under 5 years of age, only 57% of influenza‐positive cases were under 5 years of age. Eight (3%) influenza‐positive cases died. An annual seasonal pattern of influenza activity was observed. The proportion of influenza‐positive cases peaked during November–January (14–42%) in the non‐pandemic years.

**Conclusions:**

Influenza is associated with substantial morbidity and mortality in Jordan. The seasonal pattern of influenza aligns with known Northern Hemisphere seasonality. Further characterization of the clinical and financial burden of influenza in Jordan will be critical in supporting decisions regarding disease control activities.

## Background

Acute respiratory infections (ARIs) are a leading cause of morbidity and mortality worldwide,^1^ resulting in an estimated 4·25 million deaths globally each year.^2^ Mathematical modeling data suggest influenza contributes significantly to the burden of ARI morbidity and mortality, with one study estimating that 18% of all deaths due to lower respiratory infections globally in 2010 were caused by influenza.^3^, ^4^ Unfortunately, significant gaps exist in current estimates with little or no data available on influenza epidemiology in many parts of the world, including the Eastern Mediterranean region.

The Hashemite Kingdom of Jordan is a medium human development country^5^ in the Eastern Mediterranean region with an upper‐middle income economy.^6^ The population is over 6·5 million with 79% living in urban areas. The capital city Amman has a population over one million. Jordan has a Mediterranean‐style climate characterized by arid summer months with a cool rainy season from November to April.^7^ Little has been published on influenza activity in Jordan, and there is no national seasonal influenza vaccination policy for all Jordanians.

Sentinel surveillance for severe acute respiratory infections (SARI) was initiated in Jordan in 2008 in the initial stages of development of the Eastern Mediterranean Acute Respiratory Infection Surveillance (EMARIS) Network. The EMARIS Network was formed through the collaboration of Ministries of Health of Egypt, Jordan, Oman, and Qatar; U.S. Centers for Disease Control and Prevention (CDC); U.S. Naval Medical Research Unit No. 3 (NAMRU‐3); and World Health Organization (WHO) Eastern Mediterranean Regional Office (EMRO). We aim to describe the epidemiology and seasonality of influenza hospitalizations in Jordan over a time period of 6 years (2008–2014).

## Methods

### Setting and study design

**Figure 1:**
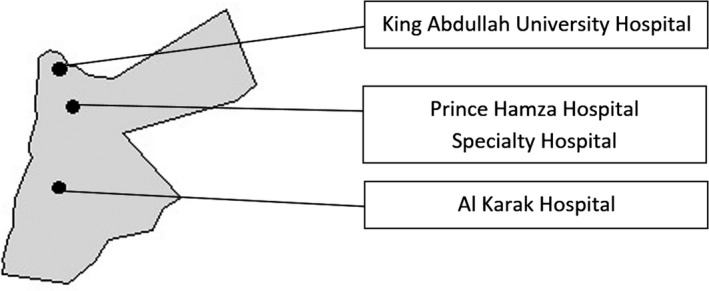
Locations of sentinel hospitals for severe acute respiratory infection (SARI) surveillance, Jordan, 2008–2014. *Locations are approximate*.

Four sentinel hospitals were selected for SARI surveillance based on being a general hospital admitting all ages, and representative geographical distribution across Jordan (Figure 1). Jordan Ministry of Health (JMOH)'s Prince Hamza Hospital and the private Specialty Hospital are large (402 and 540 beds, respectively) referral medical centers in Amman with an approximate catchment area of 2·2 million. Also included were King Abdullah University Hospital (683 beds) in northern Jordan and JMOH's Al Karak Hospital (135 beds) in southern Jordan with approximate catchment areas of one million and 170 000, respectively. The first three sites began enrolling patients in January 2008, and Al Karak Hospital in the south began enrolling patients in November 2008. All sentinel sites were general hospitals that enrolled patients of all ages. Labor and delivery services were available at both King Abdullah University Hospital and Al Karak Hospital.

Hospital surveillance teams comprised of a surveillance coordinator, internist, and laboratory director were identified and trained at each facility on case definitions, case enrollment, and data and specimen collection. Teams screened all hospitalized patients for SARI enrollment eligibility and those who met the case definition and provided informed consent were enrolled in SARI surveillance. Surveillance teams collected laboratory specimens and extracted demographic and clinical information from medical records of enrolled patients.

Eligible cases were identified using a syndromic case definition for severe acute respiratory infections (SARI). Clinical case definitions were standardized across all countries involved in the EMARIS Network. Case definitions were reviewed annually by the network and modified based on input from the partners as well as changes in WHO guidance. Consequently, case definitions evolved over the study period (Appendix A). During 2008–2009, the WHO SARI case definition (2006)^8^ was used. During 2010–2011, the WHO SARI case definition was expanded to include cases of any age ≥31 days, cases that met the CDC International Emerging Infection Program (IEIP) pneumonia case definition^9^ or any case that a clinician suspected to be a case of SARI. In January 2012, the revised WHO SARI case definition (2011)^10^ was adopted although suspect SARI cases reported by clinicians that did not meet the case definition continued to be included.

### Sample collection and laboratory procedures

One oropharyngeal and one nasopharyngeal swab were obtained from each enrolled patient when possible, although some patients may have had only one swab taken due to patient comfort. A 15‐ml tube containing both swabs (or one swab if both were not available) was agitated vigorously for 10 seconds using a vortex mixer. Each swab was removed and discarded, while the resulting supernatant in 2‐ml viral transport medium was decanted into two cryovials labeled with the patient's study identification (ID) number. These were immediately stored in liquid nitrogen and transported to the Central Public Health Laboratory (CPHL) of the Ministry of Health, also a recognized WHO National Influenza Center (NIC). Real‐time reverse transcription polymerase chain reaction (rtRT‐PCR) testing was conducted to identify influenza virus types and influenza A subtypes (no influenza B subtyping was conducted) following standard procedures.^11^ Initially, all samples were tested in parallel at the regional reference laboratory of the US Naval Medical Research Unit (NAMRU‐3) in Cairo. Starting in September 2011, after CPHL in Jordan achieved over 90% concordance between their results and NAMRU‐3 results, quality assurance testing continued on 10% of tested samples.

### Data collection and analysis

Data were collected on network‐standardized case report forms and included: age, residence, sex, date of symptom onset, date of hospitalization, and date of discharge or death. Information from medical records was used to assess chronic medical conditions including preexisting respiratory, cardiac, renal, hepatic, hematologic, neurologic, infectious, endocrine, and gastrointestinal conditions, as well as pregnancy status. Forms from each enrolled patient were collected, and the data entered at the JMOH into a Microsoft Access database (Redmond, WA, USA) organized by patients’ study ID number. Beginning in March 2010, double data entry was performed on case report forms to reduce errors. A consolidated, merged database containing all epidemiologic and laboratory data was cleaned, checked for errors, and imported to sas 9.2 (SAS Institute, Cary, NC, USA) for analysis.

Influenza laboratory results were recorded as influenza‐negative, influenza A/H1N1, influenza A/H1N1pdm09, influenza A/H3N2, or influenza B. Any specimen positive for more than one influenza result was assigned a single result for analysis according to which type ranked first according to the following hierarchy (based on perceived public health importance): influenza A/H1N1pdm09, influenza A/H3N2, influenza A/H1N1, and influenza B. Bivariate tabulations were performed on demographic and clinical characteristics by influenza laboratory result to obtain counts and percentages, and to compare categorical variables by Pearson's chi‐square test. Categorical variables of three or more levels found significant by Pearson's chi‐square test were assessed by level using binomial logistic regression. Select continuous variables such as age and the duration from symptom onset to hospitalization were described using the median, range, and interquartile range. The monthly influenza positivity rate was calculated as the percentage of specimens that tested positive for influenza out of the total specimens tested each month.

We assessed influenza seasonality by evaluating the months having peak and trough proportions of influenza‐positive specimens each year. The month having the lowest proportion of influenza‐positive specimens was defined as the nadir of influenza activity and the month having the highest proportion of influenza‐positive specimens each year was defined as the peak of influenza activity. The calendar month with the lowest average influenza activity was determined over the entire study period, and we defined the influenza season as from the beginning of the lowest average activity month in 1 year to the end of one calendar year from this date.

### Ethical considerations

Surveillance was initiated under a protocol approved by NAMRU‐3 and CDC Institutional Review Boards (IRB) and the Ministry of Health in Jordan. In 2010, SARI surveillance was adopted as part of Jordan's national influenza and communicable disease surveillance strategy.

## Results

From January 2008 to February 2014, a total of 2891 cases met a SARI surveillance case definition and all had swabbed samples tested for influenza by rtRT‐PCR. Of these, 257 (9%) were positive for influenza. A total of 192 samples (7%) were positive for influenza A, and 65 samples (2%) were positive for influenza B. Of the 192 influenza A samples, 47% were subtyped as seasonal A/H3N2, 46% were subtyped as A/H1N1pdm09, and 7% were subtyped as seasonal A/H1N1. No unsubtypeable influenza A specimens were identified. Four co‐infections of influenza A and B were recorded as influenza A for analysis (two of influenza A/H1N1pdm09 and B and two of influenza A/H3N2 and B), and no other co‐infections were detected.

The age groups of SARI patients by viral influenza result are presented in Table 1. Fifty‐seven percent of influenza cases (146/257) were under 5 years of age. Influenza‐positive patients were less likely than influenza‐negative patients to be under 2 years of age (*P* < 0·0001), and more likely to be within the age groups of 2–4, 5–14, 15–49, and 50–64 years (all *P* < 0·05). The median age of influenza cases was 3 years (range 0–85, interquartile range 0–21) compared with 1 year (range 0–103, interquartile range 0–4) for non‐influenza SARI cases.

**Table 1:** Age groups of patients enrolled in sentinel surveillance for severe acute respiratory infection by viral influenza result, Jordan, January 2008–February 2014

Characteristic	Influenza positive (*N* = 257)	Influenza negative (*N* = 2634)	*P*‐value^a^
	*N* (% column total)		
Age group^b^			<0·0001
<2 years^b^	91 (35)	1589 (60)	<0·0001
2–4 years^b^	55 (21)	384 (15)	0·0039
5–14 years^b^	31 (12)	221 (8)	0·0477
15–49 years^b^	49 (19)	204 (8)	<0·0001
50–64 years^b^	14 (5)	76 (3)	0·0265
≥65 years	12 (5)	130 (5)	0·8505

a The *P*‐value for the age group variable is from Pearson's chi‐square test, and *P*‐values were obtained for each level of the variable from binomial logit regression.

b *P* < 0·05 (predetermined statistical significance level).

Males comprised 60% of patients with influenza‐positive samples and 59% of patients with influenza‐negative samples (*P* = 0·92). Ten (4%) of those with influenza were admitted to an intensive care unit and eight (3%) died. Seven of eight (88%) influenza deaths were among children less than 5 years of age, all were among children less than 15 years of age, six (75%) were female, and four (50%) had a chronic medical condition. There were no significant differences between patients having influenza‐positive and influenza‐negative SARI specimens regarding preexisting chronic conditions (16% and 15%, respectively) and mortality (3% and 2%, respectively). Of 11 pregnant women with SARI, one (9%) had an influenza‐positive specimen and zero died. Across all SARI cases, the median duration from symptom onset to hospitalization was 3 days (interquartile range 2–4 days).

Of the 6 years of the study having available results for all 12 months, a nadir of influenza activity occurred in July and September in five of those years, more than any other month. July had the lowest influenza activity over the study period, with two influenza cases from 2008 to 2013. Consequently, the influenza season in Jordan over the study period was from July of one year through June of the following year. During the 2008–2009, 2010–2011, and 2011–2012 seasons, influenza activity peaked in the month of December, while influenza activity peaked in October in the 2009–2010 season and in January in the 2012–2012 season. In the months of peak influenza activity, influenza accounted for from 14% of all SARI hospitalizations during the 2008–2009 season to 55% of all SARI hospitalizations during the 2009–2010 season, with influenza accounting for an average of 34% of all SARI hospitalizations during the months of peak influenza activity over the study period.

**Figure 2:**
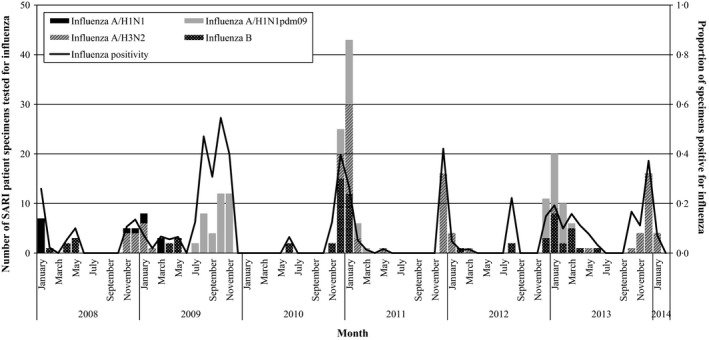
Number and proportion of severe acute respiratory infection (SARI) surveillance patient specimens positive for influenza by viral influenza etiology and month, Jordan, January 2008–February 2014 (*n* = 257).

Influenza virus types and subtypes varied by season (Figure 2). The predominant influenza virus in each season was as follows: A/H3N2 in 2008–2009, A/H1N1pdm09 in 2009–2010, B in 2010–2011, A/H3N2 in 2011–2012, and A/H1N1pdm09 in 2012–2013.

## Discussion

This study is the first systematic description of the epidemiology of influenza in Jordan. We use data from sentinel surveillance for severe acute respiratory infections in Jordan to describe the activity and seasonality of influenza hospitalizations in the country. From January 2008 to February 2014, we found that influenza was detected in 9% of all patients with SARI admitted to the surveillance hospitals, although during peaks of influenza activity influenza accounted for up to 55% of SARI hospitalizations. Those with influenza were older (median, 3 years old) than those who presented with SARI and tested negative for influenza (median, 1 year old) but did not otherwise differ in demographic characteristics. Overall, 3% of the hospitalized influenza cases died and all of these deaths were among children. The system was able to detect predominant types/subtypes of influenza during each season, including the emergence of influenza A/H1N1pdm09. Finally, the system allowed us to define the seasonality of influenza in Jordan from July through June with peaks of disease occurring in the winter months.

The influenza surveillance system in Jordan appears to provide data consistent with that found in other similar surveillance systems in other parts of the world. The proportion of influenza‐positive SARI cases (9%) compared well with that of similar surveillance systems in Asia (8%,^12^ 9%^13^) and Africa (10%,^14^ 11%^15^). In addition, the system readily detected the emergence of the 2009 influenza pandemic. Even in non‐pandemic years the predominant influenza viruses detected in Jordan correlated with those seen in similar systems.^12^, ^13^, ^14^, ^15^

Our data show that influenza has a substantial impact on health in Jordan. At the peak of activity, influenza accounted for an average of 34% of SARI hospitalizations, affecting people of all ages, with 16% of those with influenza reporting some chronic medical condition. Given that about one‐third of SARI hospitalizations are due to influenza during the peak of a given influenza season, the availability of resources such as antiviral medications during this time could be crucial. While only eight deaths were identified, almost all of these deaths were among young children under 5 years of age. Additional work is needed to define the population burden of influenza and how that burden falls on different age groups and populations. This information will be essential to policymakers in making decisions on the costs and benefits of control strategies, such as vaccination.

The seasonality of influenza we observed in Jordan matches Northern Hemisphere circulation as well.^16^ Knowledge of influenza seasonality affords public health authorities the opportunity to focus public health interventions such as education campaigns and vaccination just prior to and during periods of increased risk. In addition, the predominant influenza types and subtypes identified in Jordan matched those circulating in the Northern Hemisphere.^17^ While additional work on sequencing the circulating viruses would inform how well they matched the strains used in Northern Hemisphere seasonal influenza vaccines, the available data support the use of Northern Hemisphere vaccine in Jordan.

Initiation of surveillance for SARI and influenza has provided a number of important benefits in Jordan. This surveillance system provides a systematic and sustainable system for monitoring influenza in Jordan, thus providing the essential data to describe and monitor influenza epidemiology in the country to inform policymaking decisions on influenza control and pandemic preparedness strategies. Developing this surveillance system has helped strengthen laboratory and epidemiologic capacity, and these capacities help Jordan in achieving compliance with International Health Regulations, 2005.^18^ This system provides important data that can contribute to the Global Influenza Surveillance and Response System.^19^ In addition, this system was developed in Jordan in parallel with regional partners as part of the multinational EMARIS Network. The goal of the EMARIS Network is to provide a forum for partner countries to share experiences and develop common methods with the goal of using future data for shared research activities. Finally, this system of surveillance sites linked to advanced laboratory capacity allowed for the rapid development of surveillance for the novel respiratory pathogen Middle East respiratory syndrome coronavirus (MERS‐CoV) that has recently been described in the region.^20^ Consequently, this system of SARI and influenza surveillance clearly provides a variety of important benefits both to Jordan and more broadly to regional and international partners working toward disease control and prevention.

There are a number of limitations to our data. Our system detected hospitalized patients meeting the SARI case definition, which intrinsically limits our sample to those with more severe illness. Therefore, the results may not be indicative of milder influenza activity and trends. The ability of our system to identify cases of influenza is influenced by the sensitivity and specificity of the SARI case definition. Moreover, different case definitions were used over time, and this may have affected the sensitivity of the system over time. The consistency of our data with respect to seasonality, its comparability to other Northern Hemisphere temperate countries, and the proportion of SARI cases that are influenza positive suggest that this surveillance system is performing comparably to other SARI‐based influenza surveillance systems. In addition, the high proportion of children among those with SARI and influenza observed may have been influenced by a number of factors. This may reflect societal differences in who is brought in for care. It may also reflect biases in surveillance with better case ascertainment and enrollment among children. Other SARI surveillance systems have also found high proportions of young children,^12^, ^13^, ^14^, ^15^ so this may suggest that the SARI case definitions used might better identify pediatric symptomatology. Finally, incomplete clinical information was provided for some of our clinical variables, which may have limited our ability to examine these factors in those with influenza. Efforts are ongoing to maintain the motivation of surveillance staff to collect complete and accurate information.

Future plans for the Ministry of Health in Jordan are to use these data to establish the burden of influenza in different risk groups within the country. These data, along with the established seasonality of influenza in Jordan, will provide critical information for policy decision‐makers to assess the costs and benefits of introducing interventions to decrease the burden of influenza in the country.

## Financial support

The study was funded through the Global Emerging Infections Surveillance Program (GEIS) GEIS847705·821000·25GB.E0018.

## Conflict of interest

The authors declare no conflicts of interest.

## Disclaimer

The views expressed in this article are those of the authors and do not necessarily reflect the official policy or position of the Department of the Navy, Department of Defense, the Centers for Disease Control and Prevention, U.S. Government, nor the Ministry of Health in Jordan. Maha Talaat and Patrick Dawson are contractors of the U.S. Government. Waleed El‐Shoubary and Mayar Saeed are employees of the U.S. Government. This work was prepared as part of their official duties. Title 17 USCx105 provides that ‘copyright protection under this title is not available for any work of the United States Government.’ Title 17 USC × 101 defines U.S. Government work as work prepared by a military service member or employee of the U.S. Government as part of that person's official duties.

## Human research protections/IRB statement

The study protocol was approved by the Naval Medical Research Center Institutional Review Board in compliance with all applicable Federal regulations governing the protection of human subjects.

## Previous presentations statement

This work has not been presented elsewhere.

## Appendix A Definition, time period, and justification for three severe acute respiratory infection (SARI) case definitions used in sentinel surveillance in Jordan, January 2008–February 2014

**Table:**

	Case definition 1	Case definition 2	Case definition 3
Time period	January 2008–December 2009	January 2010–December 2011	January 2012–February 2014
Definition	2–59 months old + Hospitalized + Cough OR tachypnea + At least 1 danger sign of pneumonia^a^ OR ≥5 years old + Hospitalized + Fever (≥38°C) + Cough OR sore throat + Shortness of breath OR difficulty breathing	≥31 days old + Hospitalized + History of fever OR current fever (≥38°C) OR current hypothermia (<35·5°C) + At least 1 sign of respiratory infection^b^ OR ≥31 days old + Hospitalized + At least 1 physician assessment criterion^c^	Hospitalized + Cough AND fever (≥38°C) within the last 7 days OR Hospitalized + Clinically suspected respiratory infection
Justification	Used WHO SARI case definition (2006)^20^	Expanded criteria to include CDC/IEIP pneumonia case definitions^9^ Added physician assessment criteria to capture more cases Removed age‐specific criteria to simplify definition	Used revised WHO SARI case definition (2011)^10^ Kept clinical suspicion available Simpler definition was easier for surveillance Captured more than 95% of overall and influenza‐positive cases compared to previous definition

WHO, World Health Organization; CDC, US Centers for Disease Control and Prevention; IEIP, International Emerging Infections Program.

a Danger signs of pneumonia included nasal flaring, chest indrawing, inability to breastfeed, vomiting, grunting, convulsions, stridor, tachypnea, and lethargy.

b Signs of respiratory infection included abnormal breath sounds, tachypnea, cough, sputum production, haemoptysis, chest pain, sore throat, and dyspnea.

c Physician assessment criteria included severe influenza‐like illness, pandemic H1N1 2009, suspected or X‐ray‐confirmed pneumonia, and other respiratory illness.
